# Estimation of an Examinee's Ability in the Web-Based Computerized Adaptive Testing Program IRT-CAT

**DOI:** 10.3352/jeehp.2006.3.4

**Published:** 2006-11-22

**Authors:** Yoon-Hwan Lee, Jung-Ho Park, In-Yong Park

**Affiliations:** Department of Information and Statistics, Hallym University, Chuncheon, Korea.

**Keywords:** Item Response Theory, Computerized Adaptive Testing, Examinee's Ability, Estimation

## Abstract

We developed a program to estimate an examinee s ability in order to provide freely available access to a web-based computerized adaptive testing (CAT) program. We used PHP and Java Script as the program languages, PostgresSQL as the database management system on an Apache web server and Linux as the operating system. A system which allows for user input and searching within inputted items and creates tests was constructed. We performed an ability estimation on each test based on a Rasch model and 2- or 3-parametric logistic models. Our system provides an algorithm for a web-based CAT, replacing previous personal computer-based ones, and makes it possible to estimate an examinee's ability immediately at the end of test.

## INTRODUCTION

Although computerized adaptive testing (CAT) has been widely used for large scale examinations and for the assessment of the psychological indices, no freely available, well-documented web-based program is known. An ability estimation algorithm was first described by Baker [1] that can be used for a personal computer base. Kim et al [2] at Busan University in Korea published the java source for a CAT in a textbook first in Korean. However, nowadays the user interface for examination has rapidly changed from personal computer-based to web-based. For a CAT system, the following four components are necessary:

- Management system of the examination, including an item bank
- System of parametric estimation for the items according to item response theory
- System of estimation of examinee's ability
- System of reevaluation of items after termination of examination

There is no integrated program freely available to execute CAT in the educational fields including the above four categories. Therefore, to provide an open access web-based computerized adaptive testing (CAT) program based on the Rasch model and 2- and 3-parametric logistic models, we tried to make a web-based ability estimation program after constructing the management system of examination, including an item bank.

## MATERIALS AND METHODS

Tools used for programming were as follows:

### Server Environment

- Operating System (OS): Fedora Core 4 (Linux Kernel Version: 2.6.11)
- Web Server: Apache 2.2.0
- Database management system (DBMS): PostgresSQL 8.1.2
- Languages: PHP-5.1.2 with Zend Optimizer 2.6.2, HTML and JavaScript

### User interface

- OS: Microsoft Windows XP SP2/Linux desktop
- Browsers: Internet Explorer 6.x, Firefox 1.5.0.7

### Algorithm

The key algorithm was already known for the ability estimation [2]. It was coded for web-inferface.

## RESULTS

### Input of items

**Fig. 1:**
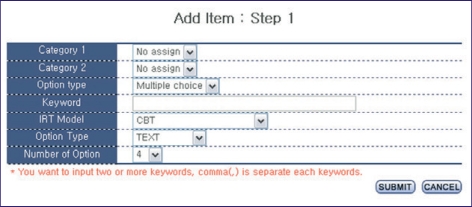
Screen shot of the characteristics of inputted items.

**Fig. 2:**
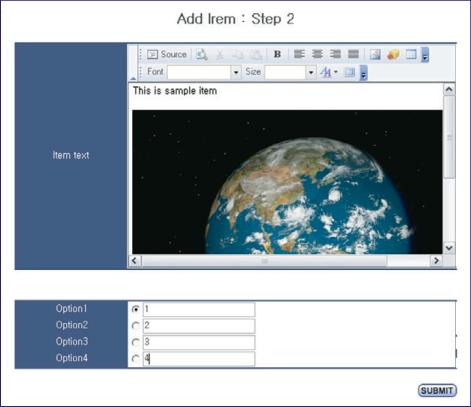
Screen shot of the input for the item stem and answer options.

**Fig. 3:**
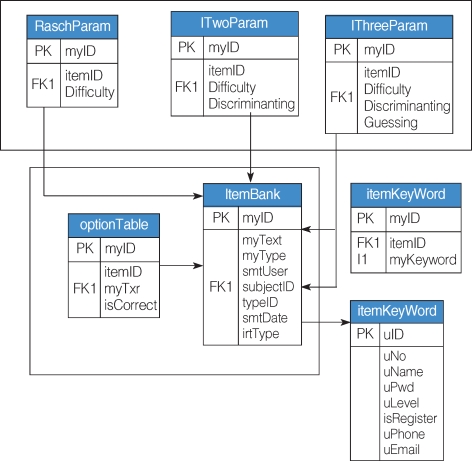
Table structure for item information.

Items can be inputted on a website. At first, basic information, such as category, item type, key words, IRT model, item parameters already determined, answer option form and number of answer option (Fig. 1), are added. An item stem can be inputted for text, and flash files can be inputted for multimedia data such as images, sounds and movie (Fig. 2). Items are presented randomly to each examinee. The table structures are shown in Fig. 3.

### Search within items

**Fig. 4:**
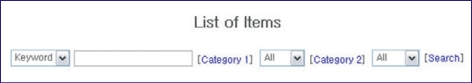
Screen shot of an item search.

**Fig. 5:**
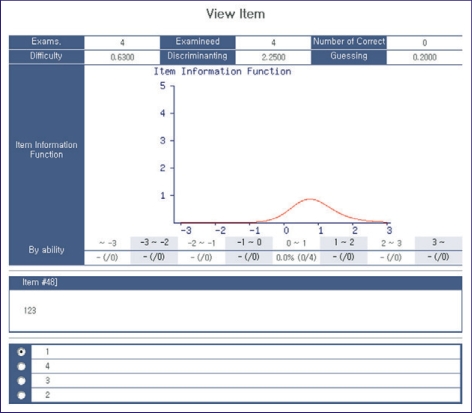
Screen shot of an item function.

On the screen, key words or text words from the items can be used to search. A Boolean search with two categories is also possible (Fig. 4). As for the CAT, each item that was already analyzed according to the respective IRT model can be searched. Each item's information function is viewed, and the rate of options by examinees in the CAT is also presented (Fig. 5).

### Creation of test and the addition of items

**Fig. 6:**
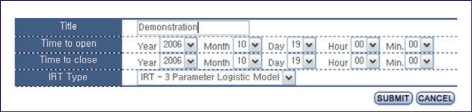
Screen shot of the creation of an examination.

To create the test, the title, start and stop time, and type of test should be inputted (Fig. 6). Available types are computer-based testing, Rasch model, and 2- or 3-parametric logistic models. The addition of items to the test can be done by selecting items in the item bank or by adding new items manually. Added items are automatically registered in the item bank.

### Estimation of the examinee s ability

**Fig. 7:**
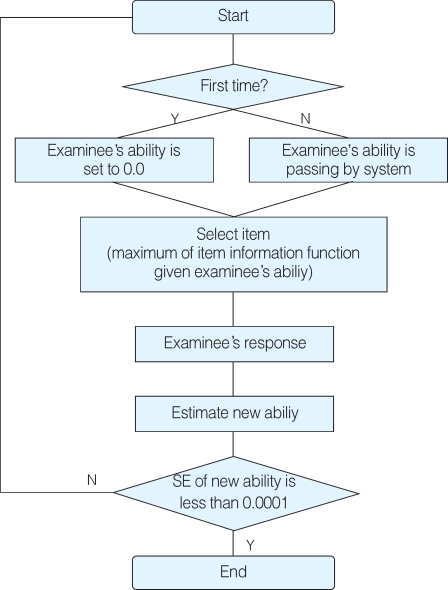
Flow chart of the ability estimation in the IRT-CAT. SE; standard error

Estimation of the examinee's ability was done according to the maximum likelihood estimation for the Rasch model and 2- or 3-parametric logistic models. The theoretical process was referenced from Kim et al[2]. Unlike Kim et al's source codes, the present source codes can estimate the ability of examinees from multiple examinations because this present one provides a common structure for item information functions, regardless of the number of examinations. Kim et al's source codes are only applicable to one examination. A flow chart of the ability estimation was presented in Fig. 7. Source codes for the estimation of an examinee's ability are presented in Appendix 1.

### Viewing the results of an examination

**Fig. 8:**
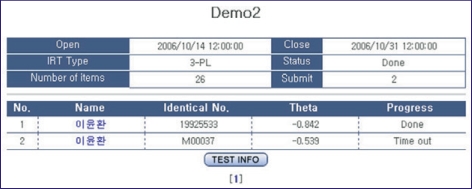
Screen shot of the examinee s ability.

**Fig. 9:**
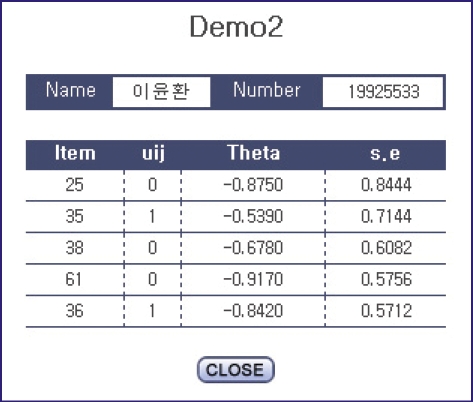
Screen shot of example of the response to an item.

After the termination of an examination, the results can be shown immediately. The examinee's ability is shown (Fig. 8). Each examinee's response to each item can be seen after clicking on an examinee's name (Fig. 9). Since the results of the examination were analyzed on the web, there is no need to download result files and analyze them again.

### CAT program

Source code of CAT in this paper is freely available at https://sourceforge.net/projects/irt-cat/

### Execution of the CAT in the field

The CAT program in this paper has already been implemented in the educational field with easy interpretations of examinees' ability [3].

## DISCUSSION

The merits of the present program for the estimation of an examinee's ability are that it is web-based and allows for the immediate estimation of ability after the termination of testing. Also, the selection of the less exposed items was prompt because the results of exposure are recorded in the system. Practical issues in developing and maintaining a CAT program included an item bank, test administration, test security and examinee issues [4]. Out of these, item bank and test administration was also actually addressed in the IRT-CAT program. Test security and examinee issues should be dealt according to the policy of each institute during the implementation of the CAT program. Since it is difficult to get a freely available CAT program, IRT-CAT may open the horizon for an easily applicable web-based CAT. It may be able to be used widely for large-scale examinations, class-based examinations, equating studies among many educational institutes, and psychological examination in the clinic. This system may still not be complete. Therefore, it will be helpful to other researchers to transform the program for their own purposes taking advantage of this program's open source code. We would like to add the system of parametric estimation for pretest items that are not used for the ability estimation according to item response theory after termination of examination to present IRT-CAT program.

## Appendix 1. Source Code of the ability estimation program

1. Input of items

// Initialize testManager 
$myIRT = new testManager($_SESSION['ID'], $_GET['examID'], $DB);
// Time setting
$myIRT->timeout(time(), $fnc->psqlTime2UT(trim($examStart)), $fnc->psqlTime2UT(trim($examEnd)));
// Set to open the examination
$myIRT->setIsTakeExam(true);
// Select item
// If the item is the first one to be shown, the examinee's ability is set to 0.0 
// If the item is the second one, set the estimated value of ability from the previous 
// item as the examinee's ability. After that, select the item with the maximum item 
// information.
$myItemID = $myIRT->selectItem();
// Initialize the class that shows the item.
$viewItem = new viewItemClass($myItemID, $DB, $_GET['examID'], "T");
// Print the test information
$viewItem->showExamInfo();
// Show item on the screen
$viewItem->showItem(1, "takeExamProc.php");

2.	Flow chart of the estimation process

// Initialize testManager
$myIRT = new testManager($_SESSION["ID"], $examID, $DB);
// Item presentation process or estimation process 
// (true : item presentation, false : estimation)
$myIRT->setIsTakeExam(false);
// item is correct or incorrect 
$myIRT->isCorrect($uij, $itemID);
// New ability estimation of examinee (transfer factor : file or DB)
$myNewTheta = $myIRT->calcTheta($isFile = true);
// Save information
$myIRT->saveData();
// Stopping rule
if( $myIRT->calcSE() ){
        require_once (MY_INCLUDE . "header.php");
        // If examination is terminated according to stopping rule, 
	 // it is terminated normally
        $myIRT->complete("Y");
}else{
        // If examination is not terminated, process returns to item presentation
?>
<SCRIPT LANGUAGE="JavaScript">
        location.href="<?=URL_ROOT?>takeExam/index.php?examID=<?=$examID?>";
</SCRIPT>
<?
}?>

3. Estimation of the examinee's ability: testManager.php

<?
class testManager {
	private $getInfoC;
	private $DB;
	private $cThetaC;
	private $sItemC;
	private $outputC;

	private $theta;
	private $userID;
	private $examID;
	private $itemID;
	private $examType;
	private $discrimination;
	private $difficulty;
	private $guessing;
		
	private $uij;

	private $uAnswer;
	private $isTakeExam;
	private $oldSE;
	private $nowSE;

	function __construct($userID, $examID, &$DB) {
		$this->DB = $DB;
		$this->userID = $userID;
		$this->examID = $examID;
		$query = "select examType from examAdmin where examID = '".$examID."'";
		$DB->query($query);
		$row = $DB->fetch();
		$this->examType = trim($row[0]);
		require_once (MY_INCLUDE . "irtManager/irtFiles.php");
		$this->getInfoC = new irtFiles($this->userID, $this->examID);

		$myLine = $this->getInfoC->readSelectLine();
		if(sizeof($myLine) < 1) {
			$this->theta = 0.0;
		}else{
			$this->theta = trim($myLine[6]);
		}
	}
	
	function setIsTakeExam($mode) {
		if($mode == true){
			$this->isTakeExam = true;
		}else{
			$this->isTakeExam = false;
		}
	}
	
	function selectItem(){
		if(!$this->isTakeExam) {
			echo "Invalid method";
			exit;
		}
		require_once (MY_INCLUDE . "irtManager/selectItem.php");
		$this->sItemC = new selectItem($this->userID, $this->examID, $this->theta, $this->examType, $this->DB);
		$myItemID = $this->sItemC->selectItem();
	
		if($myItemID > 0){
			return $myItemID;
		}else{
			$this->complete("N");
		}
	}
	function isCorrect(& $uij, $itemID) {
		if($this->isTakeExam) {
			echo "Invalid method";
			exit;
		}
		$this->itemID = $itemID;
		$this->uAnswer = $uij;
		$query = "select myID from optionTable where itemID = '".$itemID."' and isCorrect = 'Y'";
		$this->DB->query($query);
		$itemAanswerArr = array();
		for($i=0; $row = $this->DB->fetch(); $i++){
			$itemAnswerArr[$i] = trim($row[0]);
		}
		if(is_array($uij)){
			$result = array_diff($itemAnswerArr, $uij);
			if(count($uij) == count($itemAnswerArr) && count($result) == 0) {
				$this->uij = 1;
			}else{
				$this->uij = 0;
			}
		}else{
			if($uij == $itemAnswerArr[0]){
				$this->uij = 1;
			}else{
				$this->uij = 0;
			}
		}
	}

	function calcTheta($isFile = true){
		if($this->isTakeExam) {
			echo "Invalid method";
			exit;
		}
		require_once (MY_INCLUDE . "irtManager/calcTheta.php");
		$this->cThetaC = new calcTheta($this->userID, $this->examID, $this->itemID, $this->theta, $this->examType, $this->DB, $this->getInfoC, $this->uij, $isFile);
		$this->theta = $this->cThetaC->newTheta();
		$this->discrimination = $this->cThetaC->getDis();
		$this->difficulty = $this->cThetaC->getDif();
		$this->guessing = $this->cThetaC->getGue();
		$this->nowSE = $this->cThetaC->getSE();
		return $this->theta;
	}

	function calcSE() {
		if($this->isTakeExam) {
			echo "Invalid method";
			exit;
		}
		
		if($this->examType == "C"){
			return false;
		}
		$myLine = $this->getInfoC->readSelectLine();
		if(sizeof($myLine) < 1){
			$this->oldSE = 0;
		}else{
			$this->oldSE = trim($myLine[7]);
		}
		if(abs($this->nowSE - $this->oldSE) < 0.01){
			return true;
		}else{
			return false;
		}
	}

	function getOldSE() {
		return $this->oldSE;
	}

	function getNowSE() {
		return $this->nowSE;
	}

	function saveData() {
		if($this->isTakeExam) {
			echo "Invalid method";
			exit;
		}
		if(is_array($this->uAnswer)){
			for($i=0; $i < (count($this->uAnswer)-1); $i++){
				$answer .= $this->uAnswer[$i].",";
			}
			$answer .= $this->uAnswer[count($this->uAnswer)-1];
		}else{
			$answer = $this->uAnswer;
		}
		switch($this->examType) {
			case "C" :
				$query = "insert into examSubmit(examID, itemID, uID, smtAnswer, isCorrect, smtTime, takeScore) values ('".$this->examID."','".$this->itemID."','".$this->userID."','".$answer."', ".$this->uij.", now(), ".($this->theta*$this->uij).")";
				if(!$this->DB->query($query)){
					echo $this->DB->error();
					exit;
				}
				break;
			default :
				$query = "insert into examSubmit(examID, itemID, uID, smtAnswer, isCorrect, smtTime) values ('".$this->examID."','".$this->itemID."','".$this->userID."','".$answer."', ".$this->uij.", now())";
				if(!$this->DB->query($query)){
					echo $this->DB->error();
					exit;
				}
				$string = $this->itemID.",".$this->uij.",".$this->discrimination.",".$this->difficulty.",".$this->guessing.",".$this->cThetaC->getP().",".$this->theta.",".$this->cThetaC->getSE()."\n";
				$fp = $this->getInfoC->getFP("a+");
				$this->getInfoC->writeFile($fp, $string);
			break;
		}
	}
	
	function complete($status = "Y") {
		switch($status) {
			case "Y" :
				$showString = "Examination has ended.<BR><BR>";
				break;
			case "N" :
				if($this->examType == "C"){
					$status = "Y";
				}
				$query = "select sum(takeScore) from examSubmit where examID = '".$this->examID."' and uID = '".$this->userID."'";
				$this->DB->query($query);
				$myScore = $this->DB->getResult(0,0);
				$showString = "You submitted all items.<BR><BR>";
				$showString .= $this->userID."'s score is <font color='red'><B>'".$myScore."'</B></font><BR><BR>";
				$this->theta = $myScore;
				break;
			case "T" :
				$showString = " Examination is ended.<BR><BR>";
				$query = "update examAdmin set examstatus = 'D' where examID = '".$this->examID."'";
				$this->DB->query($query);
				break;
			default :
				echo "Invalid Method";
				exit;
				break;
		}
		$query = "select uid from doneExam where uid = '".$this->userID."' and examid = '".$this->examID."'";
		$this->DB->query($query);
		$uid = $this->DB->getResult(0,0);
		if($uid == "") {
			$query = "insert into doneExam( examID, uID, uResult, uStatus, smtTime) values ('".$this->examID."','".$this->userID."',".$this->theta.",'".$status."', now())";
			$this->DB->query($query);
		}
		echo $showString;
		echo "[<A HREF='".URL_ROOT."home/index.php'>List</A>]";
		exit;
	}
	
	function timeout($nowTime, $examStart, $examEnd) {
		if($nowTime < $examStart){
?>
<SCRIPT LANGUAGE="JavaScript">
	alert("It's not time to test."); 
	location.href="<?=URL_ROOT?>home/index.php";
</SCRIPT>
<?
		}else if($nowTime > $examEnd) {
			$this->complete("T");
			exit;
		}
	}
}
